# Validation of high throughput sequencing and microbial forensics applications

**DOI:** 10.1186/2041-2223-5-9

**Published:** 2014-07-30

**Authors:** Bruce Budowle, Nancy D Connell, Anna Bielecka-Oder, Rita R Colwell, Cindi R Corbett, Jacqueline Fletcher, Mats Forsman, Dana R Kadavy, Alemka Markotic, Stephen A Morse, Randall S Murch, Antti Sajantila, Sarah E Schmedes, Krista L Ternus, Stephen D Turner, Samuel Minot

**Affiliations:** 1 Department of Molecular and Medical Genetics, Institute of Applied Genetics, University of North Texas Health Science Center, Fort Worth, Texas, USA; 2 Center of Excellence in Genomic Medicine Research (CEGMR), King Abdulaziz University, Jeddah, Saudi Arabia; 3 Rutgers New Jersey Medical School, Center for Biodefense, Rutgers University, Newark, New Jersey, USA; 4 Department of Epidemiology, The General K. Kaczkowski Military Institute of Hygiene and Epidemiology, Warsaw, Poland; 5 CosmosID®, 387 Technology Dr, College Park, MD, USA; 6 Maryland Pathogen Research Institute, University of Maryland, College Park, MD, USA; 7 University of Maryland Institute for Advanced Computer Studies, University of Maryland, College Park, MD, USA; 8 Bloomberg School of Public Health, Johns Hopkins University, Baltimore, MD, USA; 9 Bioforensics Assay Development and DiagnosticsSection, Science Technology and Core Services Division, National Microbiology Laboratory, Winnipeg, MB, Canada; 10 Department of Medical Microbiology, University of Manitoba, Winnipeg, Canada; 11 National Institute for Microbial Forensics & Food and Agricultural Biosecurity, Oklahoma State University, Stillwater, OK, USA; 12 Division of CBRN Defence and Security, Swedish Defence Research Agency, Umeå, Sweden; 13 Signature Science, LLC, Austin, TX, USA; 14 University Hospital for Infectious Diseases “Fran Mihaljevic” and Medical School University of Rijeka, Zagreb, Croatia; 15 Division of Foodborne, Waterborne, and Environmental Diseases, National Center for Emerging and Zoonotic Infectious Diseases, Centers for Disease Control and Prevention, Atlanta, Georgia; 16 Virginia Tech, National Capital Region, Arlington, VA, USA; 17 Department of Forensic Medicine, Hjelt Institute, University of Helsinki, Helsinki, Finland; 18 Public Health Sciences, Bioinformatics Core Director, University of Virginia School of Medicine, Charlottesville, VA, USA

**Keywords:** Microbial forensics, Validation, High throughput sequencing, Sample preparation, Library preparation, Bioinformatics

## Abstract

High throughput sequencing (HTS) generates large amounts of high quality sequence data for microbial genomics. The value of HTS for microbial forensics is the speed at which evidence can be collected and the power to characterize microbial-related evidence to solve biocrimes and bioterrorist events. As HTS technologies continue to improve, they provide increasingly powerful sets of tools to support the entire field of microbial forensics. Accurate, credible results allow analysis and interpretation, significantly influencing the course and/or focus of an investigation, and can impact the response of the government to an attack having individual, political, economic or military consequences. Interpretation of the results of microbial forensic analyses relies on understanding the performance and limitations of HTS methods, including analytical processes, assays and data interpretation. The utility of HTS must be defined carefully within established operating conditions and tolerances. Validation is essential in the development and implementation of microbial forensics methods used for formulating investigative leads attribution. HTS strategies vary, requiring guiding principles for HTS system validation. Three initial aspects of HTS, irrespective of chemistry, instrumentation or software are: 1) sample preparation, 2) sequencing, and 3) data analysis. Criteria that should be considered for HTS validation for microbial forensics are presented here. Validation should be defined in terms of specific application and the criteria described here comprise a foundation for investigators to establish, validate and implement HTS as a tool in microbial forensics, enhancing public safety and national security.

## Background

Microbial forensics involves analysis of microbe-related materials found at a crime scene, suspected laboratory, and so on, for forensic attribution and, thus, can be pivotal for developing investigative leads. Attribution (assigning to a source) can be defined as the characterization of a sample with the greatest specificity, which in the case of a microorganism would be at the species or strain level and ideally at the level of the isolate or even the culture vessel (for example, flask) from which the sample originated. High throughput sequencing (HTS) vastly improves the possibility that the forensic and scientific communities will be able to assign features to bio-forensic evidence, such as specific identity for unknown or emerging pathogens, sample or microbe origin, antibiotic sensitivity, evidence of genetic engineering and virulence profile. Now that a number of laboratories can afford HTS systems, community-accepted validation guidelines or standards are needed. As with any analytical tool(s) for forensic application, the utility of HTS operating conditions and tolerances and interpretation guidelines must be carefully defined. Guiding principles must be established to validate HTS systems. Here we define the criteria and offer a process for validation of HTS systems in microbial forensics. If methods are validated within the framework outlined here, microbial forensics will achieve an ever higher level of power and analytical value and, ultimately, greater protection for the public and the nation’s safety and security.

## Introduction

More than a decade ago the United States experienced a simple but effective biological attack in which *Bacillus anthracis* endospores were placed in envelopes and delivered by the US postal service to intended victims [1-4]. The Federal Bureau of Investigation initiated the Hazardous Material Response Unit in 1996 to undertake a forensic investigation of bioterrorism events. Despite this effort, in 2001 the forensic infrastructure was inadequately prepared to analyze and interpret the available microbiological evidence to assist in determining who did and did not have the capacity to perpetrate such an attack. In fact, much of the needed forensic science applications had not yet been developed or validated. As part of an immediate national response to investigate such crimes, the field of microbial forensics was born [5-7] and its emergence was accelerated by the urgent requirement to investigate the anthrax mailing attacks.

The foundations of the field of microbial forensics lie in public health epidemiology and its practices [6-10] as well as agriculture practices [11-13]. Microbial forensics involves analysis of microbe-related materials found at a crime scene, suspected laboratory, and so on for forensic attribution (assigning to a source) and, thus, can be pivotal for developing investigative leads. Attribution in the case of microbial forensics can be further defined as the characterization of microorganisms within a sample to the species or strain level and ideally to the specific isolate or culture vessel from which the sample originated. Indeed, metagenomic approaches to assess microbial composition of samples also may provide strong microbial forensics evidence (either phylogenetically by identifying a specific target organism in the complex sample or by abundance spectrum profile) to attribute sample(s) to source. Scientific attribution also eliminates as many other candidate isolates or sources as possible and supports both investigation and legal proceedings.

The standards and requirements for microbial forensic practices are less well defined than those within human identification and other established forensic disciplines. However, establishing the validity of microbial forensic methods and their use and interpretation contributes to acceptance, admissibility, confidence, value and weight of physical evidence in the jurisprudence process [14] as well as within the military, intelligence and homeland security sectors that have the responsibility to act upon data and reports associated with suspected bioterror activities. Within two years following the anthrax letter attacks, the FBI’s Scientific Working Group for Microbial Genetics and Forensics (SWGMGF) formalized and published Quality Assurance (QA) guidelines [7]. The motivation for establishing a QA system was to put quality practices in place to ensure that microbial forensic evidence was analyzed using the best practices possible and that the interpretation of results was based on extant data and sound principles.

The SWGMGF QA guidelines were a good first step in establishing a QA system for microbial forensics and for increasing confidence in the data generated. However, as technologies advance and application of microbial forensics expands beyond the traditional law enforcement communities, it becomes increasingly important to continue to build upon the SWGMGF guidance and previously published microbial validation methods [7] to reflect the current state-of-the practice and foster greater community wide acceptance. Significant drivers to expand validation guidance include the substantial developments and applications of next-generation or HTS. For perspective, the first bacterial genomes that were sequenced in 1995 by the Institute of Genome Research (Rockville, MD, USA) [15,16] took more than three months to complete. Although HTS technology was initially developed, in part, for characterizing human genomes [17-19], these instruments have increasingly been used successfully to characterize unknown microbes in samples of varying complexity [20-42]. Within the field of microbial forensics [7,43,44], HTS combined with powerful bioinformatics capabilities offers a powerful tool to characterize forensic bio-evidence, including unknown microorganisms, genetically-engineered microorganisms and low-abundance (or trace) microorganisms present in complex mixed samples with extremely high sensitivity [45]. HTS technologies have features that make them more desirable and accessible for microbial forensic analyses than Sanger sequencing [46], including high throughput, reduced cost (on a per nucleotide or per genome basis) [47] and large-scale automation capability. Millions of sequencing reactions can be performed in a massively parallel fashion in a single instrument run [48-53]. With many copies sequenced at any desired portion of the genome (known as coverage), consensus sequence accuracy can be increased far beyond the per-read accuracy rate. As the throughput and accuracy of HTS continues to increase, more samples can be multiplexed in a single run without sacrificing depth of coverage or more complex samples may be analyzed at a greater depth of coverage.

Several HTS platforms are available and currently used for microbial sequencing, usually based on massively parallel sequence by synthesis strategies with high accuracy in a reduced footprint compared with Sanger sequencing. The primary HTS platforms include the HiSeq and MiSeq from Illumina (San Diego, CA, USA), the Ion PGM and Ion Proton Sequencers from ThermoFisher (South San Francisco, CA, USA) and the 454 systems from Roche (Pleasanton, CA, USA). The Illumina NextSeq 500 system is the latest platform on the market in this desktop category with 150 Gigabase throughput and ‘push-button simplicity’.

Another type of sequencing chemistry, developed by Pacific Biosciences (PacBio, Menlo Park, CA, USA), is the first to utilize single molecule real time (SMRT) sequencing, in which each base is detected in real time as a polymerase adds fluorescently tagged nucleotides along single DNA template molecules. SMRT sequencing is distinct from the other HTS technologies in providing very long read lengths. The average read length with the PacBio RS instrument is approximately 3,000 bp and can reach up to 20,000 bp [54]. Furthermore, examining the polymerase kinetics of SMRT sequencing allows for direct detection of methylated DNA bases [55]. This intrinsic capability of the SMRT sequencing workflow does not affect primary sequence determination, while yielding yet another forensic signature that is not captured with standard protocols on other HTS instruments.

A new and potentially revolutionary sequencing platform in development by Oxford Nanopore (Oxford, United Kingdom) will allow a single DNA molecule to pass through a protein nanopore set within an electrically resistant membrane bilayer. The resulting cross-membrane current fluctuations are used to identify the targeted nucleotide [56]. The company projects sequencing rates initially will be 20 bases per second, increasing to 1,000 bases per second in the future, and providing read lengths up to tens of thousands of bases [57]. While these individual reads will contain a larger number of errors than the other mentioned HTS instruments, the PacBio (and potentially the Oxford Nanopore) errors are random. With redundant interrogation of the same base of a circular template with SMRT sequencing and with sufficient depth of coverage, highly accurate consensus calls can be obtained [54].

HTS vastly improves the possibility that the forensic and scientific communities will be able to assign features (for example, strain identity, virulence profile, and so on) and, ultimately, attribution to bio-forensic evidence. However, these improvements cannot be realized or known with any level of statistical confidence without effective and validated bioinformatics tools to process, analyze and interpret the large amounts of HTS data generated. Most application-oriented laboratories are unlikely to have in-house bioinformaticians, and even for laboratories with such resources, a comprehensive data analysis pipeline must be defined and validated to establish that the software algorithm(s) reliably analyze sequence data and produce accurate final results. Many bioinformatic tools are available within commercial, academic and other open sources. However, the specific tools employed or developed are highly dependent on the need and intended use of that laboratory and may not have been rigorously tested. An appropriate data analysis pipeline must be implemented and fully validated, including understanding the uncertainty and error associated with each step of the process, as well as the collective uncertainty. The appropriate interpretation and weight of the evidence must be employed successfully and effectively communicated.

Now that laboratories are implementing HTS systems, community-accepted validation guidelines or standards are needed. Development of HTS technologies and associated bioinformatics tools will continue to progress rapidly, and, no doubt, increasingly powerful tools will be available to support microbial forensics. HTS applications for microbial forensics include assembly of draft and finished single genomes of microorganisms, targeted site sequencing, metagenomics (both amplicon sequencing of conserved genes for microbial community structure and shotgun sequencing for profiling the content of a sample), and source attribution, including profiling, sample comparison, sample engineering, and other microbial evolution or epidemiology applications. As with any analytical tool(s) for forensic application, the utility of HTS operating conditions and tolerances must be carefully defined. Regardless of the variation in technologies and software, guiding principles, such as the criteria listed in Table 1, must be established to validate HTS systems. Here we define the criteria and offer a process for validation of HTS systems in microbial forensics. Rather than delineating a set of protocols for a particular set of tools and reagents that apply to a limited set of instances, which may quickly become obsolete, those tools and reagents universally needed for protocol validation are described. By addressing each area described below, an investigator will be able to establish, validate and implement HTS as a tool for microbial forensics.

**Table 1:** Validation criteria for analytical performance metrics

**Criteria**	**Definitions**
Analytical sensitivity	Likelihood that the assay will detect a target (for example, organism variant, sequence region, functional element, and so on) in a sample (that is, target), if present; can include target attribution when defined as strain- or isolate-level detection. Also known as the true positive rate. Calculated by dividing number of true positives by the sum of true positive and false negatives (TP/(TP + FN)).
Analytical specificity	Likelihood that the assay will not detect a target, if not in the sample; can include false target attribution. Also known as the true negative rate. Calculated by dividing true negatives by the sum of true negatives plus false positives (TN/(TN + FP)). May be impractical to calculate for methods designed to detect the known universe of organisms.
Precision	The degree that individual measurements of the same sample are similar with regard to the presence and absence of target. Determined by the distribution of random errors and not the true or underlying value.
Accuracy	Degree that the material measured is similar to its true value. Calculated by (TP + TN)/(TP + FP + FN + TN).
Reproducibility	The degree to which the same result(s) is obtained for a sample when the assay is repeated between/among different operators and/or detection instruments.
Repeatability	The degree to which the same result(s) is obtained for a sample when the assay is repeated by the same operator and/or detection instrument.
Limit of detection	Minimum level of input material for a target as a proportion of the total at which all replicates are consistently positive for that target.
Reportable range	The region(s) of genome(s) that are sequenced and from which information is drawn for comparison or attribution.
False positive rate	The rate at which a target is incorrectly called as present. Also known as Type I error. Calculated as 1 – specificity
False negative rate	The rate at which a target organism is incorrectly called as absent. Also known as Type II error. Calculated as 1 – sensitivity.
Assay robustness	Stability of analytical performance under variable conditions, that is, likelihood of assay success.
Reference materials^a^	Materials/samples used to test the performance of the assay (for example, reference panels of the target and mock or non-probative materials) relevant to the intended application of the assay.
Databases^a^	Collection of data and reference genomes, genes and genomic elements to be used for interpretation of results.
Interpretation criteria for results^a^	Analysis (quantitative or qualitative) used and confidence level of a result (match, association, most recent common ancestor, and so on).

^a^These last three items – Reference materials, Databases, and Interpretation criteria – typically have not been considered validation criteria. However, they have been included here primarily because interpretation of results is an essential part of generating reliable and appropriate results, which should be described within a standard operating protocol (SOP). The data used to test a system are reliant on reference materials and, depending on the situation, databases. See [58-62].

## Application and validation of HTS for microbial forensics

Microbial forensic applications of HTS include single isolate sequencing with *de novo* assembly, read mapping, targeted sequencing of specified genes or other regions of interest (which generally include diagnostic markers, for example, SNPs, indels, and so on) [63,64], and metagenomics. Metagenomics analyzes by sequencing DNA (or RNA) samples to identify or describe microbial community composition of environmental samples such as soil [65], plants [41,42], sea water [66,67], hospital environments [68] and human-associated habitats [69,70]. HTS makes metagenomics readily feasible since culturing is not required for sample enrichment. HTS and associated bioinformatic technologies make it possible to detect microorganisms of interest when they are present in low abundance and differentiate them from near neighbors by using diagnostic genomic signatures.

Customers, stakeholders, the judicial system and the public expect forensic methods to be validated, when feasible, prior to use on forensic evidence. A validation process is essential in the development of methods for microbial forensics, and such methods must be reliable, defensible and fit for purpose.

Validation has been described as the process that:

1. Assesses the ability of procedures to obtain reliable results under defined conditions.

2. Rigorously defines the conditions that are required to obtain the results

3. Determines the limitations of the procedures.

4. Identifies aspects of the analysis that must be monitored and controlled.

5. Forms the basis for the development of interpretation guidelines to convey the significance of the findings [58].

While these general principles apply to HTS technologies and guidelines specifically for HTS used in metagenomic profiling already exist [71], there are challenges that arise when validating HTS for microbial forensics that require further consideration. Here we describe the specific guidelines for validating HTS technologies so that the microbial forensics community (and others) will have a common protocol and lexicon to leverage the exciting potential of HTS while maintaining high quality and confidence under rigorous scrutiny when this technology is used to support investigations of bioterrorism or biocrimes.

## General considerations for validation

The requirements for validation will vary according to the process in question and should be defined in terms of the specific application. While full developmental and internal validation is ideal [7,58], this requirement may not be practical for all situations, such as an attack involving a novel agent not in previously validated systems. Indeed, the use of multilocus variable number of tandem repeat (VNTR) analysis [72] to determine that the strain of *B. anthracis* in the 2001 letter attack was Ames was not a fully validated procedure in casework analysis. Yet, it was sufficiently developed for investigative lead value [73].

Because of the vast and incompletely described biological diversity of microbes and the potential of having to deal with a large number of samples in a microbial forensic case, it is not possible to validate every scenario. Moreover, HTS and bioinformatics technologies are changing rapidly and will continue to be improved in the immediate and long-range future. Lastly, exigent circumstances may require immediate response, and microbial forensics should be able to lend support using all available tools. For such unforeseen circumstances preliminary validation may be ‘carried out to acquire limited test data to enable the evaluation of a method for its investigative-lead value, with the intent of identifying key parameters and operating conditions and of establishing a degree of confidence in the methods of collection, extraction, and analysis’ [74]. However, once general validation is accomplished for instrumentation, bioinformatics data analysis, and Standard Operating Protocols (SOPs), only novel aspects of validation for new targets may be needed to generate informative leads and to make public health decisions with associated levels of confidence. Therefore, it is extremely important to establish comprehensive criteria for validation of HTS technologies with all aspects of the validation study documented. The fact that a validation study is preliminary should be stated clearly, with the limitations of the assay and validation study clearly described. However, validation of finalized SOPs is essential for reliable and defensible use of HTS technologies in microbial forensics. Sample collection and storage have been addressed elsewhere [75] and will not be described here. Validation of the HTS process addressed here relies, in part, on reports available in the literature [59-61,76] that have defined validation requirements for HTS applied to human clinical genetic analyses. The validation guidelines for the three major technical components of HTS (sample preparation, sequencing and data interpretation) as related to the field of microbial forensics, are presented in the following sections.

## Sample preparation

### Nucleic acid extraction – quantity and purity

Validation should include anticipated sample types and matrices of those sample types. A range of routinely anticipated types of samples incorporating an array of quality and quantity of nucleic acids, environmental matrices, inhibitors of downstream analytical processes and biological contaminants expected to impact reliability, specificity and obtaining results, should be included.

Template DNA (or RNA, even though DNA is referenced here) must be of sufficient quantity and quality for library preparation and sequencing. The amount of DNA available will influence the library preparation method used. At the time of preparation of this manuscript, for example, the TruSeq (Illumina, Inc.) sequencing preparation method requires approximately 100 ng to 1 μg [77], Haloplex (Agilent, Santa Clara, CA, USA) 225 ng [78], Nextera XT (Illumina) 1 ng [79], and polymerase chain reaction (PCR)-based methods, though variable, may require less than 1 ng. Minimum and maximum DNA requirements for analysis should be established using a laboratory’s work flow. A set of guidelines is needed to establish what levels of prepared DNA may be insufficient or compromised and how to proceed under such circumstances (for example, analyze anyway, stop, or select an alternate assay). Metrics based on precise quantitative pre-analytical sample characterization are needed to assess the fraction of template molecules that meet the requirements for downstream analyses, which is important for amplicon sequencing and shotgun sequencing. It is likely that samples from which the DNA is insufficient, damaged and/or inaccessible will be encountered, especially when collected from the environment. This information will be helpful to assess and compare potential downstream partial and/or complete loss of target data. The DNA extraction method used should be tested for yield and sufficient purity for downstream analytical processes. Additional extraction processes may include separating a particular genome from a metagenomic sample or selective filtration to separate specific types of microbes, such as virus particles in a metagenomic sample [71,80] or methylated DNA from non-methylated DNA [81]. Since host DNA or background genome(s) may comprise a major component(s) of a given metagenomic sample, the ability to sequence minor components of complex samples may be affected. Purification procedures used to maximize the yield of targets of interest should be evaluated the same as the nucleic acid purification process. Lastly, proper positive and negative controls should be included to assess process performance and laboratory background contamination, respectively.

### Enrichment and library preparation

DNA samples, single source or metagenomic, may be enriched for specific target regions of genomes using a capture approach or PCR. For many enrichment processes the desired genomic regions should be known and defined in order to design the protocol. However, whole genome amplification methods such as non-specific or degenerate PCR primers, [82,83] including multiple displacement amplification [84], can be used. The methods used for genome amplification can impact the results by introducing contaminating chimera formation and sequence bias [71], and should be considered, depending on the method or assay during validation.

Capture- and PCR-based methods have both advantages and limitations. PCR-based methods provide greater sensitivity of detection, but are likely to produce greater error from mis-incorporation by the polymerase than would be generated with a capture approach. PCR-based methods, in which a multiplex panel of markers may be considered, will require development of primer sets that amplify the targeted sites in a balanced fashion (or at least describe any significant imbalance) and do not cross-hybridize to unspecified targets. In contrast, capture methods will require more template DNA and would not provide the limit of detection necessary for microbial forensic analyses of trace materials. Regardless of the methods listed here or new ones subsequently introduced, it is incumbent upon the analyst to define validation criteria that address the advantages and limitations of enrichment.

Whether or not a sample is enriched, the next step in sample preparation is library preparation where the DNA sample is modified for sequencing. DNA is typically fragmented into shorter pieces by mechanical shearing (for example, sonication) or enzymatic fragmentation (for example, tagmentation [79,85]). Adapters are added to each fragment of DNA to facilitate clonal amplification prior to sequencing of the cloned fragments. Adapters can be incorporated into existing amplicon fragments during PCR. With long PCR amplicons, fragmentation may be required. DNA fragments and/or PCR amplicons then are size-selected for the range appropriate for down-stream sequencing and quality assessment. This process generates a library of millions of cloned fragments that are ready for sequencing. Quality must be assured by testing reproducibility of library preparations and robustness of indexing (described below) to identify (or misidentify) labeled fragments. Internal controls to monitor enrichment and library quality should be considered.

### Multiplexing

Multiplexing with HTS can be achieved by barcoding (or indexing) [86,87]. Short unique sequence tags are added to every fragment of a sample during library preparation to ‘tag’ the fragments unique to a sample. Thereby, samples can be pooled (or multiplexed) and data separated (that is, demultiplexed) after sequencing, based on the unique tagged sequences. With the high throughput capacity afforded by HTS, many different samples may be sequenced simultaneously. For example, the MiSeq and Reagent Kit V2 (Illumina) is capable of generating more than 7.5 to 8.5 Gbp using a 2 × 250 paired-end run (about 39 hours sequencing run time). With 8 Gbp of sequence data, 16 samples can be multiplexed on a single run assuming desired 100× coverage of a 5 Mb bacterial genome (5e^6^ bp genome × 100× coverage × 16 samples = 8e^9^ bp MiSeq output). This calculation is just an example and will change as throughput and read lengths increase, which is likely to occur relatively quickly and often. As the throughput of HTS continues to increase, more samples could be multiplexed in a single run without sacrificing depth of coverage or more complex samples may be analyzed at a greater depth of coverage. In theory, hundreds to thousands of barcodes could be synthesized, but currently 12 to 384 different reference samples can be pooled in a single reaction [86,87]). The Earth Microbiome Project provides >2,000 barcodes that could be combined, theoretically enabling multiplexing of >4 million samples in a single sequencing run [88]. Depending on the target, for example, single source samples, the number of samples that can be barcoded and sequenced in a single run should be predictable. The performance of barcoding to identify specifically tagged samples should be evaluated and documented. Furthermore, when feasible, use of different indexes in sequential sequencing runs on an instrument can indicate if carry-over contamination has occurred, which offers another quality control for monitoring potential impact of contamination on sequencing results.

## Sequencing

Each HTS system employs a unique chemistry for sequence determination and each will have to be validated in general and then specifically according to applicable features of the system [51,52,89-93]. For example, chemistries employed by 454 and Ion Torrent systems tend to be less accurate than Illumina-based chemistry for sequencing homopolymers. The type of sequencing, either single-end (fragments sequenced at one end only) or paired-end (both ends are sequenced) can impact coverage and stringency in different ways. Sanger sequencing, still considered the gold standard, allows for some concordance testing (that is, comparative analysis of the same target sequence with different analytical systems). However, there is no guarantee that the gold standard always provides the correct result. For example, Harismendy *et al*. [94] sequenced 266 kb of portions of six ion channel-related genes using Sanger sequencing, three HTS platforms, and one microarray platform and compared the results. The portion of false negative (FN) and false positive (FP) single nucleotide polymorphisms (SNPs) attributed to Sanger sequencing were 0.9% and 3.1%, respectively. Moreover, the lower throughput and coverage of Sanger sequencing makes it impractical for concordance testing with HTS generated data. The data generated by HTS are so much greater per run than those generated by Sanger sequencing that only limited sampling and very short regions can be reasonably compared. Instead concordance testing may be better achieved by testing orthogonal HTS systems with templates of ‘known’ genome sequence. Potential errors and biases inherent in each HTS system may be determined and documented better in this manner. For each sample type and platform, the error rate (and error profile) of sequencing can be determined only by empirical testing. The data can be used to define limitations of the current system that should be part of an interpretation SOP. In addition, orthogonal testing allows for identifying weaknesses and enables assay improvements before implementation. Where possible, orthogonal analyses should be employed for validating HTS methods.

## Data analysis and interpretation

**Figure 1:**
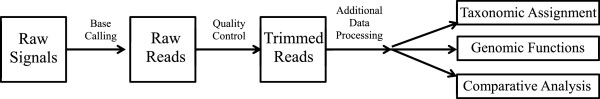
**Basic schematic of data flow through an analysis process.** The first step of base calling generally is completed by the instrument software, and each downstream step must be included in the validated analytical pipeline. Additional data processing after generating sequence reads is required, for example with contig building and/or alignment, and will depend on the application.

The final major components of HTS validation are data analysis and interpretation of results. Bioinformatics is essential and critical because of the massive amount of data, the requirement to answer forensic and investigative questions using the data, and the questions that may be addressed with trace samples, complex samples, potential genetic engineering, and background endemicity (that is, microorganisms that generally are found at a location). Comprehensive data analysis pipeline(s) should be defined and validated. It is important to establish that the software algorithms reliably analyze sequence data to produce accurate final results. The flow of data generally progresses through base calling, quality control and, finally, downstream taxonomic, functional and/or comparative analysis (which is generally either alignment- or fragment-based, if assembly is not performed) (Figure 1).

**Figure 2:**
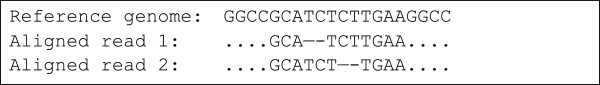
**Alternate alignments of identical sequences.** Reads 1 and 2 are aligned in equally optimal ways that indicate different locations for a 2 bp deletion relative to the reference. Differences in alignment can be problematic when an evidence sample’s consensus alignment is based on a different approach than that of the reference sample or entries in a database.

Quality metrics, generated during the analytical process, include: quality scores for base calling, read-level quality control (QC) (to include trimming of low quality bases on fragment ends), alignment, GC content, depth of coverage, strand bias and variant calling. Base calling, the identification of the specific nucleotide present at each position in a single read, should be part of instrument software. A quality threshold of base scoring is typically set with a Q score. A threshold of Q20 sets the minimum base call accuracy at 99% allowing for an incorrect base call per read at 1 in 100, while a Q30 score sets the accuracy at 99.9% and incorrect base call per read at 1 in 1,000 [95]. A Q score threshold should be set for both validation studies and subsequent implementation. However, there are no guidelines that suggest that, for example, a Q20 score is a requirement. A less than Q20 score may not impact accuracy as subsequent coverage and annotation may be adequate. Under defined conditions and for investigative leads or exigent circumstances the quality score may be relaxed; however, the justification or reliability of a lower score must be documented. Each HTS platform will have specific sequencing limitations and errors: signal-intensity decay over the read, erroneous insertions and deletions, strand bias, and so on. These limitations should be described and defined.Accuracy of identifying sequence variants (for example, SNPs, indels, chromosomal rearrangements, copy number variants) is dependent on a number of factors that include base calling and alignment as well as choice of reference genome, depth of sequence coverage (as well as average coverage), and sequence chemistry/platform. Because alignment involves arranging a read with a reference sequence (targeted or whole genome), different alignment strategies can and do produce different results (Figure 2). Differences in alignment will vary with software, so rules for alignment should be defined for consistency and traceability.

Choice of a reference genome, if used for alignment is important. Because the reference will vary by species and circumstance, specific criteria for selection are not provided here. However, it is incumbent upon the analyst to develop criteria for the reference genome that is selected. For example, from a microbiological perspective, a reference may be one that is accessible, is relevant as the Type strain, is relevant from a public health perspective, or is well-defined microbiologically; and from a computational perspective, it may be one, several or a collection of genomes, or the optimal computational sequence, and should be curated, such as a finished genome. Validation should define the purpose of the reference genome and describe the criteria for selection.

Minimum criteria should be established by the laboratory for the output of the selected analytical method, such as depth and uniformity of coverage. Defining output thresholds for metagenomic samples may be difficult given the immense quantity of data and microbial diversity; therefore, single source samples and defined mixtures can be used as a guide. These limitations may be necessary in defining FNs and FPs. Clearly, there will be ambiguous calls due to sequencing noise and novel genome composition. The specific parameters and settings used to establish thresholds, FP and FN rates should be detailed thoroughly to enable sound interpretation and accurate comparison to alternative methods and protocols.

Software may be adequate or somewhat limited with respect to the accuracy of variant calling. The limitations should be described and quantified, and algorithms may need to be modified to address specific limitations. The method(s) of identification and annotation should be described. Different formats are available for exporting variants and annotations [59]. The file format should include ‘a definition of the file structure and the organization of the data, specification of the coordinate system being used, e.g., the reference genome to which the coordinates correspond, whether numbering is 0-based or 1-based, and the method of numbering coordinates for different classes of variants, and the ability to interconvert to other variant formats and software’ [59].

The FP and FN rate often are determined for most analytical assays. However, there are additional considerations with HTS and microbial identification. Similar to homologous regions and pseudogenes for human genetic variation testing [60], the homologous regions of a near neighbor (for example, genes shared across the bacterial kingdom) become important for target identification (target being the species or strain of interest). Generating a metagenomic profile at the resolution of the phylum level, or even the genus level, may indicate a general environment from which a sample originates but often cannot identify the microorganism of interest at the species or strain level. However, newer approaches have started to achieve strain level identification [96-99] by exploiting higher throughput and novel algorithms. The results can be accurate and reliable and can translate into identification of the target agent in an unknown complex sample. Many reliable reads of the sequence of any particular species will share the same sequence, particularly so with near neighbors. For example, while *Bacillus* species may be sufficiently diverse to discriminate in a particular assay, strains of *B. anthracis* are nearly indistinguishable from one another [100]. FPs must be defined by specificity and the ability to phylogenetically differentiate a species (or strain) from near neighbors, such as *Bacillus anthracis* and *Bacillus cereus*. Testing that a known single source sample fits in a phylogenetic schema is not the same as identifying a particular species in a simple or complex sample. Methods for identification of targets should be validated based on intended use. FN rate may be difficult to determine for metagenomic sample analyses as stochastic effects and sampling variance may impact detection of the target(s). Known data sets can be helpful to define the FN rate.

Once assay conditions and pipeline configurations have been established, the entire method should be tested prior to use. Although individual components may have been validated, it is imperative to demonstrate that valid and reliable results are obtained when the components are combined. The standard microbial forensics validation criteria [7,58] apply to HTS methods as well. Special attention should be given to accuracy, precision, analytical sensitivity and specificity, reproducibility, limits of detection, robustness, reportable range, reference range, either FN/FP or confidence, statements of findings and databases used (Table 1). The laboratory must select and be able to clearly and defensibly state the parameters and thresholds necessary to determine whether the overall sequencing run is of sufficient quality to be considered successful. Criteria should include error rate, percentage of target captured, percentage of reads aligned, average and range of coverage depth, and so on.

### Reference materials

Reference materials (RMs) should be used during test validation. Well-characterized reference samples should be included to establish baseline data to which future test modifications also can be compared [60]. Many different types of samples can serve as RMs for HTS, including characterized DNA derived from specimens prepared from microbial cultures, samples collected from several different endemic regions with high incidence of microorganisms of interest, samples from several non-endemic regions discovered accidentally and described as isolated outbreaks or findings, synthetic DNA (sets of sequences of known isolates), or electronic data (that is, generated *in silico*). A gold-standard reference genome would contain a single gap-less sequence for each chromosome or plasmid in the source organism, with no ambiguous bases. RMs are homogeneous for single source samples, stable and defined. Because complex mixtures are likely to be unstable and subject to stochastic effects, simple mixtures should be used. *In silico* complex samples, which can be considered stable, are suitable for testing the bioinformatics pipeline. The sequences used and parameters employed for testing should be documented. The same rationale can be applied to positive controls, which must be defined. Negative controls may include no-template controls, blank controls for different phases of the analytical process or DNA samples void of the target.

### Bioinformatics software management

The bioinformatics community has not yet defined uniform guidelines or protocols for benchmarking software. Thus, users must fully validate and document their bioinformatics pipeline. Software may be open source, purchased from commercial entities, developed in-house, or come from a combination of sources. The software programs should perform general quality metrics assessment, but the software likely will differ in performance and potentially yield different results. Therefore, accurate versioning of the state of the software is essential [76], not just for validation but also for data analyses. The software and modifications must be tracked. Settings that can be modified by the user should be documented. Documentation also should include the specific version(s) of each component of the pipeline, the hardware, dates of use and changes to software. Each software upgrade requires revalidation of the steps downstream of HTS. Virtual Machines [101], which are software simulation(s) of a machine, encompass the entire computational environment used for analysis and can help accomplish comprehensive version control on this complete system. By maintaining informative curated reference datasets, validation of updates or changes to software pipelines may be facilitated without any additional HTS or with only minimal effort.

Analysis by computer software is an essential component of using HTS data. Two general criteria addressing software performance are verification and validation. According to the Institute of Electrical and Electronics Engineers (IEEE) Std 610.12-1990 [102], verification is ‘the process of evaluating a system or component to determine whether the products of a given development phase satisfy the conditions imposed at the start of that phase,’ and validation is ‘the process of evaluating a system or component during or at the end of the development process to determine whether it satisfies specified requirements.’ Since verification applies to whether the software system was built correctly and validation is whether the intended use was met, most users will only have tools at their disposal to perform a validation of software. To be considered validated, software must be tested using input data that replicate challenging real-world situations. Software can be integrated within the HTS process (for example, instrumentation) for data generation and external to the HTS system for interpretation (for example, phylogenetics, attribution, and so on). The software specifications should conform to the intended use [103]. Performance characteristics must define the entire process addressed by the software and individual process steps, as appropriate. Much of the above discussion on data generation addressed the criteria that relate to performance of data generation and subsequent interpretation and, thus, serves as a guide for software performance characteristics. Users who create software for intended applications should refer to the standards by the IEEE [102]. However, the majority of users in the application-oriented laboratory will obtain commercially-available software, and so validation likely will be at the ‘black box’ level. Even without access to the source code, users still are responsible for ensuring that the software performs the intended functions correctly. Regardless, since software requirements often are derived from overall system requirements for the HTS analytical tool, a systems approach is recommended for validation. The user’s intended use and needs [103] drive the criteria for validation. When possible, the user can rely on the manufacturer’s data for some of the validation, if the data are made available, or on the scientific literature. Nevertheless an internal validation demonstrating that performance criteria are met is required. Software is different than instrumentation in that it does not wear out [103], and likely will be modified for better performance over the lifespan of its use (however, the computer operating system can change, rendering the software incompatible with the newer system). Since software will be modified and updated, a validation analysis should be conducted for the specific change(s) following the same principles of validation. New problems may arise with the intended update and, therefore, any impact that modification may have on software beyond the change should be tested using a systems approach [103].

### Data storage

Permanent storage of all raw HTS data is not practical as the raw data are exceedingly large. After base-calling, this information is routinely discarded. Sequence data should be in conventional, widely used format(s), for example, .fastq files for sequence reads, or be readily convertible to a standard format [59]. Many bioinformatics pipelines create output data structures that may be compressed and stored as an alternative to the .fastq read files. Other compression systems have been proposed for HTS data [104,105], but there may be utility in widely used methods like gzip compression that will likely remain in use for years to come. A best practice should be to create a process so that results can be re-analyzed as necessary when updates are made to the downstream bioinformatics systems. Other files to archive include: SAM/BAM (mapping data) and vcf (variants). These or similar formats could be used to store alignments and data about known SNPs of special diagnostic power, such as canonical SNPs. Where possible, DNA samples should be stored for re-testing [76]. Because of limitations with large amounts of data, it is necessary that reference datasets are documented and maintained in order to perform validation of future software updates. Lastly, conversion of data from one format to another could create unforeseen transcription errors; therefore, defined data sets should be tested before and after data conversion for accuracy.

### Interpretation and reporting

Interpretation of results for attribution should be defined clearly and documented. Equally important, the level of resolution possible with a particular system should be stated. Also, the database(s) used for validation (and for casework analysis) is likely to be expanded and improved with HTS technologies on a relatively rapid basis; so the records of the database(s) used for individual analyses must be maintained. The target areas that define a species or strain and resolve it from near neighbors are critical [100,106]. One or more sites may be required depending on phylogenetic resolution. A minimum number of targets and degree of confidence with the number of targets should be established [107]. The means by which a software pipeline determines attribution may not be accessible to the user, in which case all relevant output data and associated thresholds should be documented and stored in a standard way according to the SOP. The minimum number of reads is essential for limits of detection, stochastic effects, and FNs and FPs and should be defined empirically for obtaining a reliable result(s). An interpretation statement(s) and degree of confidence (qualitative or quantitative) should be developed regarding attribution of the sample, and that confidence, when feasible, should be based in a rigorous statistical framework.

Resequencing assembly can be effective if the reference dataset contains sequences of closely related reference genomes [71]. *De novo* sequencing is computationally more demanding. Thus, the software and, just as importantly, reference data sets are critical to result quality. There are a number of assembly algorithms that can take millions of short reads generated by HTS and translate them into a portion or complete genome sequence [108-112]. Each approach has benefits and limitations affecting quality and efficiency. Therefore, specific software used, standard metrics (for example, N50, coverage, contig sizes) assumptions and criteria applied should be documented [113,114].

While there may be some situations in which assembly is required, it is less likely to be used or even necessary for the foreseeable future in microbial forensics analyses and especially with mixtures or metagenomic samples where near neighbors, strains and coverage constraints reduce the practicality of assembly. Alignment strategies or fragment counting strategies are preferable to assembly. Sequence alignment compares DNA sequences (although it can apply to any kind of sequence data) and seeks to identify regions of homology. More often a reference sequence will be maintained, targeted short reads will be aligned with that reference, and differences with respect to the reference will be listed (as ‘variants’) [115-117]. In addition to the same documentation requirements for assembly strategies, the reference sequence should be fully documented. While we believe that alignment strategies will be favored over assembly strategies for metagenomic microbial forensic applications, if capabilities improve that enable effective assembly, then it is incumbent upon the user to validate the process.

#### Taxonomic assignment

Methods for read-based taxonomic classification of metagenomics data fall into two broad categories: composition-based and alignment-based. Composition-based approaches rely on comparing signatures of short motifs from a query fragment to a reference genome -- for instance, a particular GC content, gene and protein family content, or k-mer frequency and distribution [71]. Composition based approaches include Phylopythia [118], PhylopythiaS [119], Phymm [120], the Naive Bayes Classifier [121], Sequedex [122], the Livermore Metagenomic Analysis Toolkit (LMAT) [97], GENIUS [96] and Kraken [99]. Alignment-based approaches compare reads to a set of labeled reference genomes using a basic local alignment search tool (BLAST)-based approach. Alignment based approaches include MEGAN, Bowtie, MetaPhlAn, MetaPhyler, CARMA, WebCARMA, IMG/M, MG-RAST, and others [98,116,123-132]. Additionally, methods for direct taxonomic classification of sequencing reads use a combination of both composition and sequence similarity approaches, such as MetaCluster [133], Rapid Identification of Taxonomic Assignments [134], and PhymmBL [127,128,135]. A more comprehensive review of sequence classification methodology and software is presented elsewhere [136].

Many programs use a phylogenetic approach to classify sequences and summarize results by taxonomic group. A sequence(s) can be assigned at any level from the phylum down to the species and strain. The output of the program may potentially assign a sequence(s) to any taxonomic level. Most commonly, a program will summarize the overall abundance of each taxonomic level it detects. If a species is detected and no other higher resolving sequence data are available, then strains within that species cannot be resolved based on that sequence data. Many programs may achieve assignment to the genus level, but not to species level attribution. Some programs conduct classification down to either genus or species, while other programs will assign to a variety of levels depending on the level of specificity of the input data. Programs designed to make assignment at the strain level for bacteria will need to be validated for that level of specificity as well as congruency with genus and species level summaries. Viral strain assignment poses additional challenges, as some viruses (for example, RNA viruses) can have high rates of mutation and form quasi-species for which no clear reference genomes are available [107]. Bacterial and virus level assignments are likely to improve as the number of sequenced microbial genomes continues to increase. Since phylogenetic assignments are based on extant data, the databases and software (and version) used to perform the phylogenetic analyses should be documented.

Software typically is run with thresholds for assignment likelihood that can be set at either the initiation of analysis or at the time of interpretation of output. The thresholds used for analysis should be defined and documented thoroughly. Documentation should include the step(s) at which thresholds are specified, either by user input, within configuration files, in output interpretation, or at any other step in the analytical process. Thresholds should not be assumed to be equivalent between programs or within different versions of the same program, as every step of the analysis can impact the odds or strength of assignment. While many thresholds for taxonomic assignment are set automatically, the user has a responsibility to design experiments that test the impact of thresholds on the output of known samples on taxonomic assignment and set those thresholds accordingly.

#### Abundance levels

The most basic measure of the abundance of an organism in a sample is binary abundance (that is, presence/absence). Proportional abundance provides information on a continuous scale, but usually does not accurately convey relative level of abundance. The current state-of-the-art is generally composed of abundance measures with no associated confidence values [97,122,127]. Because of the complex nature of metagenomic samples a stochastic threshold (or minimum abundance threshold) for detection should be implemented. The abundance threshold can be set empirically to where anything above that value is present and anything lower (below the limit of detection) is either inconclusive, not detected, or absent, but then should be used consistently to measure corresponding error rates. The degree of accuracy is tied to the threshold of detection that is set. Internal standards are useful. Most studies to date have collected metagenomic data in a relative framework, in which abundance of genes or messages is calculated as percent or proportion of the sample content. However, the abundance level can be more accurate if internal genomic DNA is added at the sample processing stage. If these control molecules are mixed into and processed alongside the sample-derived nucleic acids, more effective quantification and inter-sample comparisons may be performed. Internal controls also may provide information on the extent or directionality of changes in any particular gene or organisms present. For example, in tracking a particular source of a contamination, measuring a gradient pointing towards the source may be useful. When drawing a conclusion that the presence of a microorganism is, for example, inconclusive or absent, it should be stated as being below the limit of detection that is determined both by the amount of sequence data and the parameters at which the analysis program was benchmarked.

#### Organism classification

Taxonomic classification of bacteria can sometimes create the misconception that microbial species are discrete and unique entities. Rather, some species are extremely closely related to each other and may form a continuum that is not readily resolved, while others are extremely distant from other microorganisms and can be categorized effectively [106]. Unfortunately, some separately named strains have almost identical genomes, while others are more dissimilar than some pairs of species. Therefore, when evaluating the power with which genomics can be used to distinguish between and among microorganisms and, thereby, define attribution under the circumstance of the analysis (for species to strain level identification or for determining similarity between two or more samples), it is important to understand the level of genomic similarity that they share (with known diversity of extant isolates). Also, the diversity in sequence within a species should be appreciated.

When constructing a test dataset for benchmarking, a decision first must be made regarding the level of genomic discrimination required. The level of genomic discrimination will likely be based on a list of known microorganisms of interest compared to their near neighbors. Whether that value is 5%, 1%, 0.1% or less, the microorganisms used for thresholding must have degrees of similarity consistent with that threshold. When calculating the similarity of two genomes, there are at least two methods that could be used: 1) calculating the similarity of regions and genes that are shared, or 2) normalizing that alignment value to the proportion of each genome that can be aligned. The second approach may account for plasmids or horizontally-transferred elements that may distinguish two strains of the same species. However, those strain-specific genes or regions may not provide any added discriminatory power to an algorithm depending on how it is constructed. One approach may be the percent identity of common (shared) genes or regions to characterize the similarity of different genomes, so that the relationship of strains with a high degree of similarity within the core genome is not confounded by the presence of mobile elements. The performance of an algorithm should be presented only in the context of the degree of similarity between the organisms used for validation, with probability estimate, if possible.

Another strategy for selecting microorganisms for benchmarking is to use specific microorganisms that are of particular interest. For example, discriminating between a threat agent (such as *B. anthracis*) and a close relative (such as *B. cereus*) may be a higher priority than discriminating between all known species that are differentiated by at least 1%. It is important to note that such a specific target approach cannot be applied to benchmarking studies of other microorganisms as they may not, and likely will not, have a comparable level of genomic dissimilarity. The documented goal(s) of the user will determine whether the validation is designed to assess global similarity measures or the similarity of specific target organisms to their near neighbors.

#### Community structure

In addition to containing many different microorganisms, whether the same ones or very different ones, metagenomic samples will differ dramatically according to the relative abundances of microorganisms comprising the sample. Abundances of each microorganism (or taxonomic level of resolution) will vary widely, so that performance will be judged across orders of magnitude. It is difficult to predict how the presence of one microorganism may modulate the detection of another (due to similar elements in those genomes and power of discrimination of the bioinformatic method). The relative abundances of each organism can be varied across a number of replicates if the method lacks discriminatory power. This evaluation is performed best *in silico*.

The output data from a series of validation tests should consist of a set of records containing:

1. Microorganism (or taxonomic level resolved).

2. Known abundance, for example, controls.

3. Measured abundance (either proportional or binary).

4. If possible, a confidence measure (or qualitative/quantitative statement).

Sets of independent tests and repetitive tests will allow for summary statistics to be applied for assessing attribution capabilities, as well as the performance of the analytical system as a whole. Since empirical data generation is demanding and costly, the use of simulation data is strongly recommended. Power testing also can be defined, based on the number of samples to be analyzed. Comparisons of abundance values of microbes in two or more samples may be used for potentially indicating association [137]. Relevant to such analyses may be population genetic quantities, such as alpha and beta diversities [138]. The appropriate criteria for abundance distributions and comparisons should be established during validation. Current software may perform such data analyses to a degree and it is anticipated that novel programs will become available.

Rates of FPs and FNs are important measures and correspond to the sensitivity and specificity of the assay. If a proportional abundance measure is given, an abundance threshold should be set to render an interpretation of presence/inconclusive/absence. If a confidence measure is given, a more stringent threshold can be used along that dimension as well. Threshold values are dependent on the parameters of the sequencing run, as well as the program used and reference database. A validation process that establishes confidence values for a particular set of output data will only be applicable to other samples that are processed on the same platform, using the same settings (read length, and so on), filtered and processed with the same Q-score cutoffs, and then analyzed with the same taxonomic assignment program run with identical settings. This process is extremely important because the results of the validation process cannot be extended directly to an analysis in which any of those parameters have been changed or do not match.

The accuracy of proportional abundance can be measured with a correlation coefficient, either parametric (for example, Pearson) or nonparametric (for example, Spearman). Pearson’s test could indicate how closely the absolute values generated resemble the known composition, while Spearman’s test could indicate how closely the generated rank-order of each organism resembles the known composition. The utility of a program in determining the proportional abundance of individual microorganisms within a sample depends on the value of the correlation coefficient with data for controls included in the analysis. However, for many forensic applications the relative abundance of an organism is far less important than the presence or absence of that organism, along with designation to the strain level of identification. Nevertheless, for applications in which relative abundance is to be reported with confidence, thorough validation must satisfy all requirements of a binary presence analysis, with the added dimension of the correlation coefficient.

## Standard operating protocols or procedures

All validated assays require SOPs, which must be based on the results of validation that encompass all appropriate aspects of the assay process, including but not limited to: sample collection and storage, nucleic acid extraction, enrichment, library preparation, sequencing, data analysis and interpretation of results. SOPs for implementation of HTS as a diagnostic tool include: (1) standardization; (2) optimization; (3) validation; and (4) automation [139]. These concepts, while initially developed for HTS-based microbial clinical diagnostics, apply equally to developing HTS SOPs for microbial forensics. Standardization, in this context, requires selecting a set of methods, software and workflows, along with setting thresholds for making a forensic interpretation based on features present in the HTS data set. SOPs themselves must be validated, ideally with blinded prospective studies using static data analysis workflows. Finally, data analysis and interpretation SOPs ideally should be fully automated, if possible, to reduce user-configurable parameters to a minimum [139].

## Conclusions

Conveying confidence in a test or process is essential in microbial forensics because the consequences are serious and the conclusions must be based on data and resultant interpretations of evidence in the case of a biothreat event. Therefore, the limitations of methods used in microbial forensics to generate results must be reliable and defensible and the process(es) of validation will contribute substantially in defining confidence associated with an assay, method, or system. HTS is an invaluable tool, expanding the scope and power of microbial forensics to provide protection against and response to attacks with biological agents. The HTS process was described in some detail herein so that analysts, who are not experienced researchers, will have guidance on the features and criteria that should be addressed during a validation. An outline of the HTS validation criteria is provided in the list of elements below. The reader may consider such validation quite challenging. However, similar demands have been in place for forensic human identification and the benefits to that forensic science community outweigh the task of validation. It is difficult to lay out the highest priority or near-term goals here as these may vary with the test or application and, therefore, such decisions are left to the community of users. To accomplish a validation the investigator should develop criteria as he or she requires for each situation. However, the criteria and the results and conclusions from validation studies must be available for inspection by appropriate parties.

### List of elements to consider during validation of HTS for microbial forensics^a^

I. Sample Preparation

a. Template (DNA or RNA) quantity and quality

i. Minimum and maximum requirements

ii. Guidelines for action when these values fall out of range

b. Enrichment

i. Desired genomic regions for enrichment

ii. Limitations of the chosen method (for example, introduces known bias, increases error) and specific circumstances for its justified use

c. Library preparation

i. Quality, sensitivity, reproducibility and robustness of library preparation method(s) across expected sample types

d. Multiplexing

i. Performance of barcoding to identify specifically tagged samples

II. Sequencing

a. System features

i. Platform (if feasible, multiple orthogonal platforms)

ii. Chemistry

iii. Quality metrics

iv. Limitations

1. Error

2. Signal-intensity decay

3. Erroneous insertions/deletions

4. Strand bias

5. Potential for carry over contamination

III. Data analysis

a. Bioinformatics pipeline

i. Functions

ii. Quality metrics

1. Variant/sequence identification

2. Q score

3. Coverage

4. Error

5. Allele call (SNP state, indel state, and so on)

6. Threshold

7. False positive and false negative rates

iii. Reference standard

1. Variant calling

2. Gene or functional element assignment

iv. Alignment- or composition-based software

1. Functions

2. Rules for alignment

v. Phylogenetics software

1. Functions

b. Bioinformatics software management

IV. Controls

a. Level of acceptable characterization

b. Intended use

V. Reference materials

VI. Databases

VII. Interpretation

a. Sample type

i. Single source

ii. Complex or metagenomic

1. Abundance

iii. FP and FN rates

b. Attribution

i. Taxonomic assignment

ii. Association

iii. Reverse engineering

1. Sample preparation

2. Genetic engineering

iv. FP and FN rates

c. Quantitative/Qualitative statements

i. Confidence

VIII. SOPs

a. Sample preparation

i. Extraction

ii. Enrichment

iii. Reverse transcription (if necessary)

b. Library preparation

c. Sequencing

d. Bioinformatics pipeline

i. Data analysis

ii. Data storage

iii. Data transfer

iv. Interpretation

^a^It is not possible to generate an all-inclusive element list because of the wide diversity of samples, sample types, chemistries, platforms, and bioinformatics for which HTS methods may be applied. Therefore, this outline serves as a guideline, rather than an exhaustive or prescriptive regulation. The user should evaluate these elements, select those that apply, justify why some elements were not applied, and add any elements that are method specific and not included in this outline.

The HTS validation process should, at a minimum: 1) ensure that appropriate reference and benchmarking datasets are used to establish FP and FN values within a rigorous statistical framework; and 2) require the practices, chemistries, settings, and bioinformatics programs used to generate actionable results be thoroughly documented and standardized, at least within the specific laboratory employing the method(s). It is important to remember that identification to species and strain is highly dependent on phylogenetic similarity of near neighbors used for comparison. Consequently, the validation of a process to detect a given species or strain cannot be applied indiscriminately to additional target organisms without additional validation. The ability of a process to identify to species level varies across the tree of life, and validation processes must take the phylogenetic framework into consideration.

The validation process described herein allows for translation of research tools to forensic applications so that HTS can provide the reproducibility and specificity necessary to stand up to the full weight of legal scrutiny. In addition to validation, the laboratory is urged to adopt an overall quality management and quality assurance system to provide a working foundation essential for microbial forensics, maintaining good laboratory practices and bolstering confidence in results.

As HTS technologies continue to advance, costs will continue to drop, instruments will become faster, smaller, eventually portable, and their applications continue to increase. Advanced sequencing technologies will begin to be applied to measuring DNA modifications, epigenetic factors and offer yet another layer of specificity. With longer reads, genetically engineered organisms will be detected, most likely by identification of unexpected adjacency of genomic elements. The validation criteria described in this paper may likely apply to the new analytical flourishes in the coming years and, therefore, provide a stable foundation for future implementation in microbial forensics. If methods are validated within the framework outlined here, microbial forensics will achieve an ever higher level of power and analytical value and, ultimately, greater protection for the public and the nation’s safety and security.

## Abbreviations

bp: base pair; FN: false negative; FP: false positive; HTS: high throughput sequencing; IEEE: Institute of Electrical and Electronics Engineers; PCR: polymerase chain reaction; QA: quality assurance; RMs: reference materials; SMRT: single molecule real time; SNPs: single nucleotide polymorphisms; SOPs: standard operating protocols; SWGMGF: Scientific Working Group for Microbial Genetics and Forensics.

## Competing interests

The authors declare that they have no competing interests. Ethics: No IRB approval was required for this work.

## Authors’ contributions

BB and SM conceived and developed the topic and wrote the majority of the manuscript; NC, AB, RC, CC, JF, MF, DK, AM, SAM, RM, AS, SS, KT and ST provided input and review. All authors read and approved the final manuscript.

## Financial disclosure

The affiliated primary institutions and agencies supported this study by providing salaries for the authors. The funders had no role in study design, data collection and analysis, decision to publish, or preparation of the manuscript.
